# 3D printed *in vitro* tumor tissue model of colorectal cancer

**DOI:** 10.7150/thno.52450

**Published:** 2020-10-26

**Authors:** Haoxiang Chen, Yanxiang Cheng, Xiaocheng Wang, Jian Wang, Xuelei Shi, Xinghuan Li, Weihong Tan, Zhikai Tan

**Affiliations:** 1 College of Biology, Hunan University, Changsha, Hunan, 410082, China.; 2 Shenzhen Institute, Hunan University, Shenzhen, Guangdong, 518000, China.; 3 Department of Gynecology, Renmin Hospital of Wuhan University, Wuhan 430060, China.

**Keywords:** biofabrication, disease modeling, tumor-associated cells, tumor microenvironment, 3D tissue

## Abstract

**Rationale:** The tumor microenvironment (TME) determines tumor progression and affects clinical therapy. Its basic components include cancer-associated fibroblasts (CAFs) and tumor-associated endothelial cells (TECs), both of which constitute the tumor matrix and microvascular network. The ability to simulate interactions between cells and extracellular matrix in a TME *in vitro* can assist the elucidation of cancer growth and evaluate the efficiency of therapies.

**Methods:** In the present study, an *in vitro* 3D model of tumor tissue that mimicked *in vivo* cell physiological function was developed using tumor-associated stromal cells. Colorectal cancer cells, CAFs, and TECs were co-cultured on 3D-printed scaffolds so as to constitute an extracellular matrix (ECM) that allowed cell processes such as adhesion, stemness, proliferation, and vascularization to take place. Normal stromal cells were activated and reprogrammed into tumor-related stromal cells to construct a TME of tumor tissues.

**Results:** The activated stromal cells overexpressed a variety of tumor-related markers and remodeled the ECM. Furthermore, the metabolic signals and malignant transformation of the *in vitro* 3D tumor tissue was substantially similar to that observed in tumors *in vivo*.

**Conclusions:** The 3D tumor tissue exhibited physiological activity with high drug resistance. The model is suitable for research studies of tumor biology and the development of personalized treatments for cancer.

## Introduction

Colorectal cancer is currently considered a cancer predominant in both men and women 1, 2. Thus, it is imperative to develop an *in vitro* model of colorectal cancer that can be used to study tumor progression and develop novel therapies against it. Tumors are characterized by disorganization and chaotic tissue formation that occurs in organs, in which the stroma co-exists and co-evolves with cancer cells 3. The tumor microenvironment (TME) of a solid tumor is a complex, interstitial extracellular matrix containing a variety of stromal cells, including fibroblasts and endothelial cells that are recruited from surrounding tissues 4, 5. Cancer-associated fibroblasts (CAFs) have been shown to regulate multiple aspects of tumorigenesis and promote the growth, survival, and spread of tumors via improved functionality and modification of the secretome 6. CAFs can also enhance angiogenesis by secreting factors that activate endothelial cells and pericytes 7. Tumor-associated endothelial cells (TECs) are involved in tumor malignancy and metastasis 8, 9. Abnormal TECs can cause a disordered vascular tumor microenvironment, thus affecting not only the metabolism of tumors 10 but also their resistance to drugs 11. Understanding the physiology of a tumor within a specific TME should therefore allow that TME to be used as a treatment target 12, 13.

Various tumor models, including patient-derived cancer cells (PDCs) and patient-derived xenografts (PDXs), have been developed which have contributed significantly to cancer research 14. However, as the mechanisms of tumor progression and the tumor microenvironment have become elucidated, the limitations of the models have become increasingly apparent. Traditional two-dimensional (2D) cultures lack the diversity of internal spatial information, cell types, and the TME 15. Although animal models can simulate physiological conditions and reflect interactions between various systems, clinical trials have had a low rate of success, requiring a long culture cycle, and are associated with high costs 16. Microtissues or organoids are independent research tools derived from three-dimensional (3D) culture technology 17. Patient-derived organoids can better maintain the characteristics of primary and tumor cells in long-term culture than can PDCs or PDXs 18. Although organoids have many advantages over traditional models and *in vitro* models of tumors, they are unable to completely replicate the complexity and diversity of primary cells and lack elements of the immune system, key stromal cells, and vascular factors 19. Therefore, the development of novel 3D tumor tissues that can be used as a tumor model for preclinical studies is highly desirable.

Three-dimensional bioprinting is a promising and versatile technique that can improve the level of reproducibility and standardization of 3D tumor models 20. In colorectal cancer research, the use of 3D printed tissue models is still relatively uncommon, mostly conducted with scaffold-free organoids. Organoids have already been demonstrated to be suitable for studying normal and pathological processes as throughput platforms 21, 22. However, they are not suitable for mimicking cell-cell and cell-ECM interactions that can affect the efficacy of anti-cancer drugs 20. Thus, there remains a lack of standardized methods and techniques for the manipulation, in addition to validation, of different 3D models and their standardization for scale-up 23. Co-cultures with tumor-associated stromal cells that use of tumor ECM as a scaffold material have significantly promoted the utilization of 3D models to study tumors 24, 25. However, the majority of relevant cells and ECM are obtained from *in vivo* sources, possibly causing instability of the observed response 26. Research studies have shown that the co-culture of tumor cells with TME-associated cells is a novel approach for characterizing various aspects of the TME 27. Due to their intrinsic plasticity, TME-associated cells derived from normal cells can be transformed into tumor-specific stromal cells through regulation by tumor signaling 28, 29 and can be developed into an *in vitro* 3D tumor model 30. In the present study, we developed a conditioned culture methodology for obtaining tumor-associated stromal cells and established a reproducible 3D colon cancer tissue model (3DT) consisting of three cellular components: colorectal cancer cells, CAFs, and TECs. The model was constructed using 3D-printed scaffolds, allowing the direct interaction between cells and the formation of a tissue network structure. The 3D tumor model also displayed a physiological state similar to that found *in vivo* and had high drug resistance, and was compatible with continuous monitoring and functional evaluation of long-term culture.

## Methods

### Materials

Type I collagen (average molecular weight (M_n_) = 2-4 × 10^3^ Da) was obtained from Shanghai Ryon Biological Technology Co. Ltd. (Shanghai, China). Polycaprolactone (PCL; M_n_ = 1.2 × 10^5^ Da), sodium alginate, and calcium gluconate were purchased from Sigma-Aldrich (USA). N,N-dimethylformamide (DMF) was acquired from the Chinese Medicine Group (Beijing, China). 40,6-diamidino-2-phenylindole (DAPI), Alexa Fluor 568 phalloidin, Calcein AM, and propidium iodide (PI) were all obtained from Yeasen Biological Technology Co., Ltd. (Shanghai, China).

### Fabrication of 3D scaffolds

**Figure 1:**
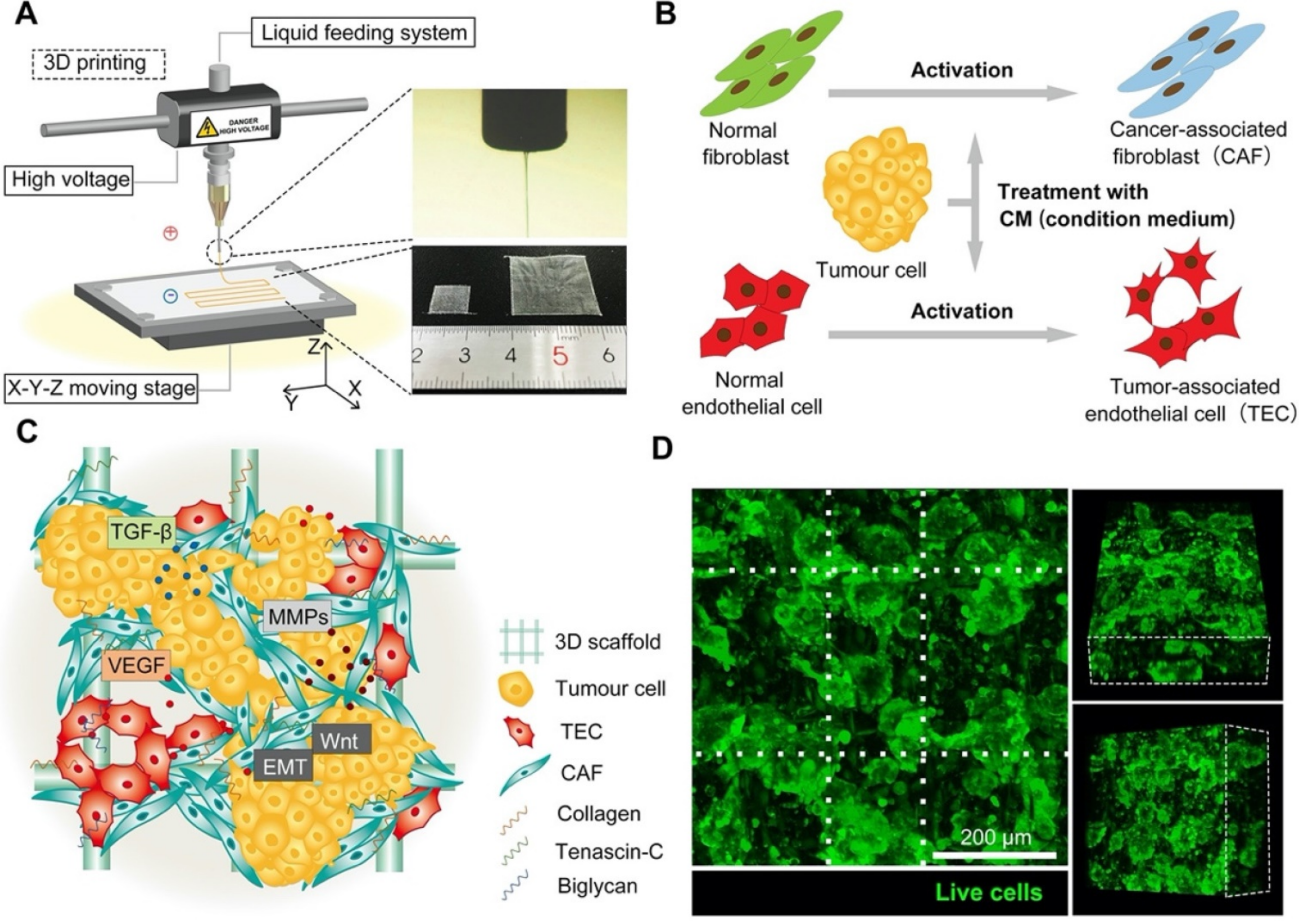
** Construction and characterization of the *in vitro* 3D tumor tissue. A)** Schematic illustration of the E-jet 3D printing device. **B)** Flow chart of cell activation pathways. **C)** Illustration of the 3D tumor tissue. **D)** Fluorescence images of the tumor tissue (green = live cells, scale bar = 200 µm). White dotted lines indicate the center of the scaffold fibers.

The 3D scaffolds used in the present study were manufactured using a custom-built electro-hydrodynamic jet (E-jet) 3D printing system consisting of a 3D collection platform, a liquid feed system, a high voltage power supply, and an observation system (Figure 1A) 31, 32. The system used an electric field to induce fluid flow from micro nozzles to print fibers within the micro/nano-scale range. To prepare the printing solution, PCL pellets were dissolved in DMF then stirred at room temperature for 24 h. The solution (10%, w/v) was then supplied to a blunt-ended needle (27G, ID: 0.21 mm, OD: 0.41 mm) in the 3D printing system at a flow rate of 0.18 mL/h. The printing solution was charged by applying a high voltage (2.6-3.2 kV) to the tip of the needle. Various PCL scaffolds were prepared by controlling the movement of the collection platform and use of a thermostatic hot plate to facilitate solvent (DMF) evaporation and scaffold formation.

The collagen solution was prepared by dissolving 0.1 mol of acetic acid in 1 L of water, into which 1 g of collagen powder was added. The resultant solution was shaken on an oscillator for 15 min until the collagen was completely dissolved. The mixture was subsequently filtered through a 0.22 μm filter for sterilization. The 3D-printed PCL scaffolds were immersed into the collagen solution for 24 h and then air dried. Similarly, petri dishes immersed in collagen solution and dried were used as a control.

### Cell culture

A human colorectal cancer cell line (HCT116), and two types of normal stromal cell, including human umbilical vein endothelial cells (HUVECs) and human embryo lung fibroblasts (HELFs), were obtained from the American Type Culture Collection (ATCC). Cells were cultured in petri dishes (2D culture) or on scaffolds (3D culture) in Dulbecco's Modified Eagle's Medium (DMEM) supplemented with 10% fetal bovine serum (FBS). Cell viability was measured using a calcein-AM/PI (Live/Dead) double staining kit (Yeasen, China). To ensure that cell morphology was preserved, serum-free medium (rather than the buffer provided in the kit) was used as a solvent to dissolve the calcein-AM (at 1:1000) and PI (at 3:1000). Cell imaging and photographing were conducted using an Olympus confocal laser scanning microscopy (CLSM, FV1000, Japan).

### Cell activation

To obtain TME-associated cells, normal stromal cells (HELFs and HUVECs) were activated and transformed into CAFs and TECs, respectively. Firstly, conditioned medium (CM) derived from HCT116 cells (denoted HCT116-CM) was prepared. The CM consists of the supernatant following cell culture. When the cells were 80% confluent, the medium was replaced with fresh medium (DMEM + 10% FBS + 1% penicillin-streptomycin (P/S)) after which the cells were incubated for 24 h. The cells were then centrifuged, and the supernatant (HCT116-CM) was collected and filtered through a 0.22 μm filter. To culture HELFs and HUVECs, the cells were initially grown in normal medium (DMEM supplemented with 10% FBS) then allowed to adhere to the petri dish or scaffold, as appropriate. After rinsing with PBS to remove any residual medium, the normal medium was replaced with HCT116-CM medium (100%) to enhance activation efficiency. After 72 h, the cells became activated and transformed into CAFs or TECs, after which they were analyzed by quantitative gene analysis to assess the degree of activation.

To examine how TME-associated cells regulate tumor cells, CM was collected from CAFs and TECs, then added to tumor cell (HCT116) cultures. Briefly, after TME-associated cells (CAFs and TECs) reached 80% confluence, the medium was replaced with fresh medium (DMEM + 10% FBS + 1% P/S) for 24 h. The supernatant containing CM was then collected by centrifugation and filtered through a 0.22 μm filter. To subculture the HCT116 cells, after adherence of the cells to petri dishes or scaffolds, the normal medium (DMEM supplemented with 10% FBS) was removed and the cells were rinsed with PBS to remove any residual medium. The cells were then cultured in a mixture of 50% CAF-CM (or TEC-CM) and 50% normal medium. After 72 h, the tumor cells were collected and analyzed by quantitative gene analysis to determine the effect of TME cells on tumor cells. The CM from normal stromal cells (HELF-CM and HUVEC-CM) was also collected and used as a control.

### 3D culture and the generation of tumor tissues

The 3D-printed scaffold was spread out in a culture dish and sterilized by UV for at least 1 h prior to use. In routine cell culture, tumor cells (HCT116) were seeded onto scaffolds and cultured in DMEM supplemented with 10% FBS. The tissue formation process was evaluated using a live/dead staining assay. To investigate scaffold universality (i.e., whether they promote tissue formation), HCT116, CAFs, and TECs were co-cultured in parallel. Briefly, the three cell types were mixed at a ratio of 5:1:1 (depending on the histological features of the tumor tissues (Figure S7) and the reference of tumor modeling 11), seeded onto sterile scaffolds then cultured in DMEM supplemented with 10% FBS.

Cells cultured in collagen-alginate hydrogels were used as the control. Sodium alginate (1%, w/v) was mixed with collagen solution (2 mg/mL) in phosphate buffer saline (PBS), which was then filtered through a 0.22 μm filter prior to use. The cell suspension (2×10^5^ cells/mL) was vigorously mixed with collagen-alginate solution (volume ratio = 1:1) to final concentrations of sodium alginate, collagen and cells of 0.5% (w/v), 1 mg/mL and 1×10^5^ cells/mL, respectively. The mixture was then added dropwise into calcium gluconate solution (1%, w/v) and then allowed to stand for 5 min until hydrogel spheroids were formed. The spheroids were subsequently transferred to a petri dish containing DMEM supplemented with 10% FBS medium and used for long-term culture.

To construct the 3D bio-printed tumor tissue (3DT), the collagen-PCL scaffold was placed in a petri dish (10 cm in diameter) and sterilized under UV light for 1 h. Activated stromal cells (CAFs and TECs) were mixed with tumor cells (HCT116) at a ratio of 5:1:1 (HCT116:CAF:TEC) and then seeded (1×10^6^ cells/mL) onto the 3D scaffolds 11. The scaffolds were placed in an incubator for 2-4 h with 2 mL DMEM to avoid uneven cell seeding caused by scaffold floating. After the cells had adhered to the scaffolds, 8 mL of medium were added. From days 0 to 5 (early stage), the medium was changed every 2-3 days, and tissue formation was assessed daily. After 5 days (late stage), when the cell number has increased dramatically, observations were conducted more regularly, and the medium replaced daily. Cells cultured for 7 days were analyzed by RNA sequencing, after which the tumor tissue was stored for further use.

### Integrin inhibition

Assessment of the activations of the stromal cells cultured on 3D scaffolds or 2D petri dishes was conducted by inhibition of integrin signals on the cells. The integrin inhibitory peptide Arg-Gly-Asp-Ser (MedChemExpress, China) was dissolved in HCT116-CM at a concentration of 250 μg/mL 33. Normal stromal cells were seeded on the scaffold already seeded with a mixture of HCT116-CM and integrin inhibitory peptide. The 3D collagen-PCL scaffolds were placed in a petri dish (10 cm in diameter) and sterilized by UV light for 1 h. After HELFs were seeded on the scaffold (1 × 10^6^ cells/mL), 2 mL of medium (DMEM + 10% FBS + 1% P/S) were added (to avoid uneven cell seeding caused by scaffold floating). The petri dish containing the scaffold was then placed in an incubator for 2-4 h. After the cells had adhered to the scaffold, the medium was replaced with HCT116-CM medium containing Arg-Gly-Asp-Ser, and the cells were cultured for a further 72 h. In addition, cells were cultured in a 2D petri dish in normal medium as a blank control. Cells were cultured on the 3D scaffold in HCT116-CM medium without inhibitory peptide as a positive control.

### Quantitative real-time PCR and agarose electrophoresis

The expression of genes involved in stromal cell activation, tumor wingless-related integration site (Wnt), and epithelial-mesenchymal transition (EMT) was evaluated by quantitative real-time PCR (qRT-PCR). The primer sequences used for qRT-PCT are detailed in Table S1 (supplementary information). Cells were digested with trypsin (Solarbio, Beijing, China) and rinsed with PBS. RNA was then extracted from the cells using RNAIso Plus (Takara, Japan) in accordance with the manufacturer's instructions. The cDNA was synthesized from the RNA using a PrimeScript RT reagent kit (Takara, Japan), and qRT-PCR conducted using TB Green Premix Ex Taq II (Takara, Japan). Expression levels of target genes were normalized to that of the housekeeping genes GAPDH or β-actin. Untreated HELFs, HUVECs, or HCT116 cells served as controls.

The expression of genes in the control group, possibly not expressed or with extremely low expression, was analyzed by agarose gel electrophoresis. The qRT-PCR products were mixed with 6x loading buffer (Takara, Japan) and then electrophoresed on a 2% agarose gel. The gels were imaged using a 4200SF gel imaging system (Tannon, China).

### Immunofluorescence analysis

Cell proliferation, adhesion, stemness, activation, and tumor cell heterogeneity were characterized by immunofluorescence staining. Briefly, the specimens were fixed in 4% paraformaldehyde for 15 min at room temperature, after which they were rinsed with PBS then permeabilized using 0.1% Triton X-100 for 10 min. The specimens were then incubated with primary antibodies, including anti-vinculin (1:100, Bioss, bsm-6640R), anti-CD133 (1:100; ABclonal, A12711), anti-KI67 (1:50; Proteintech, 27309-1-AP), anti-BAX (1:100; Proteintech, 50599-2-lg), anti-BCL2 (1:100; ABclonal, A0208), anti-vimentin (1:100; ABclonal, A2666), or anti-E-cadherin (1:100; Proteintech, 60335-1-lg), at 4 ℃ for 12 h. After rinsing with PBS, the specimens were incubated with secondary antibodies, including FITC-AffiniPure goat anti-mouse IgG (H+L) and Cy3-AffiniPure goat anti-rabbit IgG (H+L) (Yeasen, China) at room temperature for 3 h in the dark. They were then rinsed with PBS. Cell nuclei were stained with DAPI, after which the cells were imaged using an Olympus confocal laser scanning microscope (CLSM, FV1000, Japan).

### Assessment of VEGF secretion

HUVECs or TECs were seeded into the wells of 12 well culture plates (2×10^5^ cells/mL) in 2 mL culture medium. Firstly, cells were cultured with complete DMEM medium for 24 h, then rinsed three times with PBS and grown in FBS-free DMEM medium to exclude the influence of serum. After 24 h, the supernatant was collected and filtered through a 0.22 μm filter to remove dead cells and cell debris. An enzyme-linked immunosorbent assay (ELISA kit, Andygene, China) was used to measure the secretion of VEGF, in accordance with the manufacturer's instructions.

### RNA Sequencing

The engineered 3D tumor tissues (3DT) were analyzed by RNA sequencing. As a control (2D-co), HELFs, HUVECs, and HCT116 cells were co-cultured in a petri dish and collected when the cells had reached an appropriate confluence. The 3DT was also digested and the resultant cell suspension was then injected subcutaneously into immunodeficient mice. After 3 weeks, the colorectal cancer tissue (CT) was harvested. Total RNA was isolated from the 3DT (or 2D-co/CT) using TRIzol reagent (DP424, TIANGEN TRNzol Universal Reagent, China), in accordance with the manufacturer's instructions, and genomic DNA degraded using DNase I (Invitrogen, USA). The quality of the RNA was determined using an Agilent Bioanalyzer 2100 (Agilent Technologies, Inc., USA). RNA concentration was measured using an ND-2000 (NanoDrop Technologies, USA). The mRNA was isolated using oligo-dT beads (Vazyme, N401, VAHTS^®^ mRNA Capture Beads, China) and digested in fragmentation buffer. Short mRNA fragments were used as a template for the synthesis of first-strand cDNA using random hexamer primers, and second-strand cDNA synthesized in response buffer containing dNTPs using DNA polymerase I. The double-stranded cDNAs were purified using AMPure XP beads (Vazyme, N411-01, VAHTS® DNA clean Beads, China) and then used for end repair and “A” base addition, and thereafter ligated with sequencing adapters.

The adaptor-ligated fragments with different sizes were purified using AMPure XP beads. After quantification using a Qubit 2.0 fluorometer (Life Technologies, USA), the cDNAs was used for PCR amplification and sequenced as 2×120 bp paired-end reads on an Illumina HiSeq^TM^ 2000 sequencer (Illumina, San Diego, CA, USA). The significance of differences between log-fold changes of differentially expressed genes (up- or down-regulated) in cells from the 3DT, 2D-co, and CT were determined, using a threshold of *p*<0.05. Analysis of biological pathways was conducted using the Database for Annotation, Visualization, Integrated Discovery (DAVID) 34, an information resource database.

### Drug resistance test

Three common chemotherapy drugs, including five-fluorouracil (5-FU), cisplatin (CDDP), and doxorubicin (Dox), were used to test for drug resistance. Cancer cells (HCT116) were divided into three groups: (1) HCT116 cells alone cultured in a 2D petri dish (2D-HCT116); (2) HCT116 cells alone cultured on the 3D scaffold (3D-HCT116); and (3) HCT116 cells from the co-cultured 3DT. The dosages of the drugs were 25 μM (5-FU) 35, 30 μM (CDDP) 36, and 10 μM (DOX) 37. Cells were treated with drugs for 24 h and were then assessed using a live/dead assay and immunofluorescence staining.

### *In vivo* experiments

Immunodeficient mice (BALB/C, 4 weeks old), purchased from Hunan SJA Laboratory Animal Co., Ltd. (Changsha, China), were used in the animal experiments. All animal experiments were approved by the Animal Experiment Ethics Committee of Hunan Experimental Animal Center (Permit No.: SYXK (Xiang) 2018-0006). Cells in the 2D-co and 3DT groups were used for the tumorigenesis experiment, conducted to assess differences in physiological function. To ensure that the numbers of cells were the same, cells in the 3DT were digested. Cells (1.5 × 10^6^ cells/mL) from both the 2D-co and 3DT groups were injected subcutaneously. The size of the tumors was measured regularly during tumor growth, volume calculated as follows:


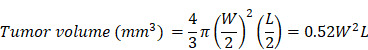


where *W* and *L* represent the width and length of the tumor, respectively.

### Statistical analysis

All statistical analyses were performed using GraphPad Prism (v7, GraphPad Prism Software, Inc., USA). Data were analyzed using Tukey's honest significant difference test. Each experiment was repeated at least five times and the results were presented as means ± SD. Differences in which *P*-values <0.05 were considered significant.

## Results

### 3D scaffolds promote growth and adhesion of cells

We sought a suitable model to construct a niche-like milieu for tumor cells *in vitro*. 3D-printing technology (Figure 1A) combined with the inherent plasticity of cells (Figure 1B) was utilized to construct a TME for organ-specific cancer tissue (Figure 1C). The 3D scaffolds exhibited an orderly spatial structure that provided excellent physical support for cell growth (Figure S1, Supplementary Information). Through activation, stromal cells became tumor-like niche cells that finally formed a 3D tumor tissue *in vitro* (Figure 1D). We also produced scaffolds with different gaps between the fibers and found that cell proliferation depended on the size of the gap - cell proliferation was greatest when gaps between the fibers were approximately 150 μm (Figures S2A & B). Additionally, the cell proliferation rate reached a peak when the aspect ratio of the micro-grid was 2:1 (300 μm:150 μm) (Figures S2C & D). The elastic modulus of the scaffold decreased as pore size increased (Figure S2E). The thickness of the scaffolds used in each experiment was 500 μm.

**Figure 2:**
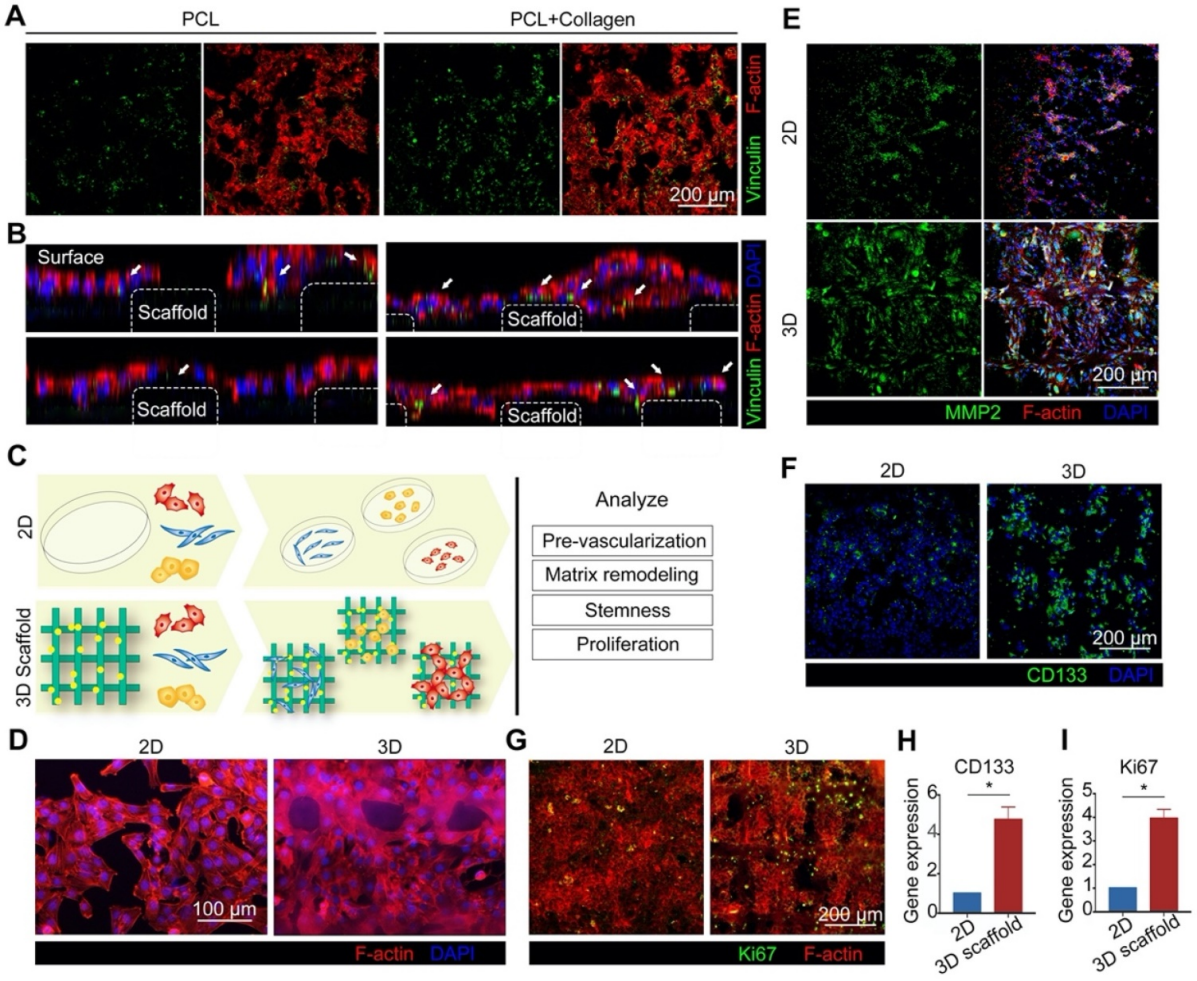
** 3D scaffolds promote a diversity of cell function. A)** Immunofluorescence staining of adhesion marker vinculin (green) in HCT116 cells cultured on 3D collagen-coated and uncoated scaffolds (scale bar = 200 µm). **B)** Localization of vinculin in (A). The dimensions of each scaffold were 1 cm × 1 cm × 500 µm. **C)** Schematic representation illustrating physiological changes to cells grown in 2D and 3D environments. **D)** Cell morphology of HUVECs grown in 2D and 3D environments (red (phalloidin) = F-actin, blue (DAPI) = nuclei, scale bar = 100 µm). **E)** Immunofluorescence staining of MMP2 (green) in HELFs grown in 2D and 3D environments (scale bar = 200 µm). **F)** Immunofluorescence staining of stem cell marker CD133 (green) in HCT116 cells grown in 2D and 3D environments (Scale bar = 200 µm). **G)** Immunofluorescence staining of proliferation marker Ki67 (green) in HCT116 cells grown in 2D and 3D environments (scale bar = 200 µm). **H-I)** Gene expression of CD133 (H) and Ki6 (I) in HCT116 cells grown in 2D and 3D environments (*p < 0.05). All values are expressed as means ± SD, n = 5.

We further observed that the 3D collagen-modified scaffold promoted cell adhesion, as indicated by HCT116 cells with additional pseudopodia (Figure S3). Vinculin is a component of the cytoskeleton and a focal adhesion protein, playing an important role in cell adhesion, extension, movement, proliferation, and survival 38. To characterize the strength of cells adhesion to the collagen-modified scaffolds, the expression of vinculin in HCT116 cells was assessed by immunofluorescence staining, and found that cells grown on the scaffolds displayed increased vinculin expression (Figures 2A & B). We also found that vinculin was located mainly at the interface between scaffold and cells (Figure 2B), indicating that the 3D collagen-scaffold promoted the adhesion of cells, thereby resulting in greater cell proliferation. Thus, the 3D scaffolds exhibited great potential for tissue constructions.

### 3D scaffolds induce cell phenotypes

There is no doubt that 3D scaffolds provide a multi-dimensional adhesion environment. However, the effect of the 3D scaffolds on cells is not only limited to adhesion, but it is also significant for other physiological functions of cells, such as pre-vascularization, matrix remodeling, stemness, and proliferation (Figure 2C). Compared with a 2D environment, the 3D scaffold displayed better performance for the induction of cell phenotype, indicating that the 3D scaffold represented a better environment for the growth of tumor cells, and thus more suitable as an *in vitro* tumor tissue model. The vascularization of endothelial cells plays an important role in the pre-vascularization of tumor tissue *in vitro*. The results demonstrate that HUVECs cultured on the 3D scaffolds are more likely to form circular structures (Figures 2D & S4), possibly induced by the morphology of the scaffolds. Matrix remodeling caused by matrix metalloprotein 2 (MMP2) secreted by fibroblasts is also important in angiogenesis 39. Compared with the 2D-cultured HELFs, the expression of MMP2 in 3D-cultured HELFs was greater (Figures 2E & S5). Furthermore, collagen deposition was apparent on the 3D scaffolds (Figure S6).

3D scaffolds are not only better at inducing stromal cells, but also tumor cells. The results demonstrate that HCT116 cells grown on the 3D scaffolds expressed more stem cell marker CD133 compared with those grown in the 2D environment (Figures 2F & H). Cancer stem cells (CSCs) represent the state at which tumor cells begin to adapt to the microenvironment prior to development into their final cell phenotype 40, 41. The environment of the 3D scaffolds up-regulated the expression of stem cell markers in HCT116 cells, causing the tumor cells to change into CSCs. In addition, CSCs can be characterized by specific markers and their function. Because CSCs have self-renewal capability, thus exhibiting infinite proliferation, we measured the cell proliferation-related protein Ki67, a proliferation marker protein of colon cancer cells. The results indicate that the expression of Ki67 (Figures 2G & I) in a 3D environment was greater than in a 2D environment, suggesting that tumor cells grown in a 3D environment have a higher proliferation rate.

### Neoplastic reprogramming of stromal cells

**Figure 3:**
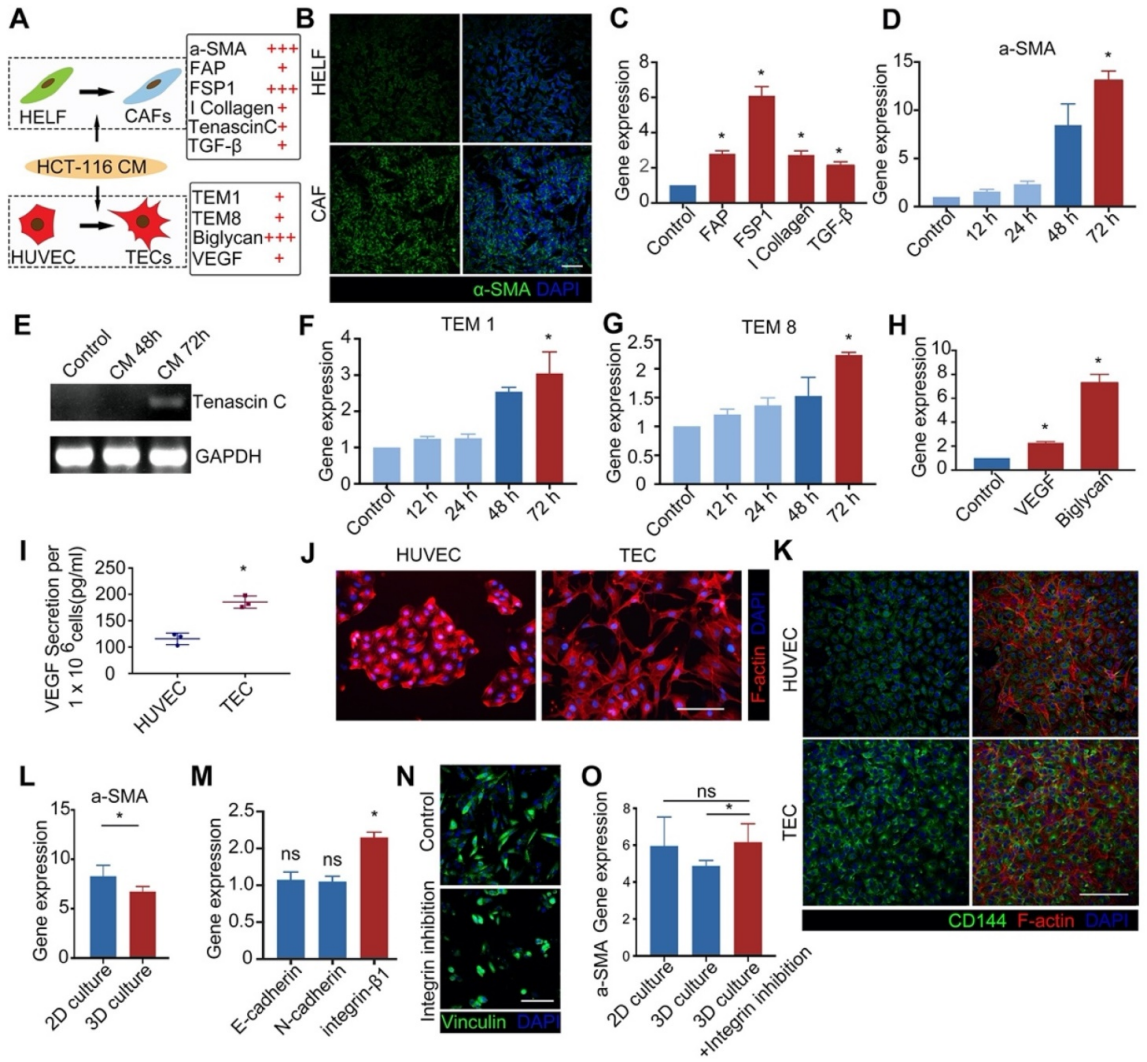
** Activation of stromal cells *in vitro*. A)** Cell activation pathway. Normal stromal cells (HELFs and HUVECs) were cultured in conditioned medium from tumor cells (HCT116-CM), and markers of cell activation were measured. + indicates >2-fold upregulation of gene expression, +++ indicates >6-fold upregulation of expression. **B)** Immunofluorescence images of HELFs and CAFs (green = α-SMA; blue (DAPI) = nuclei) (scale bar = 100 µm). **C)** Expression of genes encoding FAP, FSP1, collagen I, and TGF-β in fibroblasts activated with CM for 72 h. **D)** Expression of α-SMA in fibroblasts treated with CM for 0-72 h. **E)** Expression of the *tenascin C* gene in activated CAFs (but not in normal HELFs). Cells in the control group in C, D & E are HELFs. **F-G)** Expression of activated marker genes TEM1 (F) and TEM8 (G) in HUVECs treated with CM for 0-72 h. **H)** The expression of genes encoding VEGF and biglycan in HUVECs activated with CM for 72 h. Cells in the control group in F, G & H are HUVECs. **I)** ELISA of VEGF secreted by HUVECs before and after activation (TECs). **J)** Morphology of HUVECs before and after activation (TECs) (red (phalloidin) = F-actin, scale bar = 100 µm). **K)** Phenotype of HUVECs before and after activation (TECs) (CD144 (green), scale bar = 100 µm). **L)** Expression of α-SMA in HELFs cultured on collagen-coated Petri dishes (2D environment) and 3D collagen-PCL scaffolds (3D environment) 72 h after treatment with HCT116-CM. **M)** Expression of E-cadherin, N-cadherin, and integrin-β1 in HELFs cultured on 3D scaffolds for 48 h. **N)** Characterization of cell adhesion after inhibition of integrin (scale bar = 100 µm). **O)** Expression of α-SMA in HELFs cultured on collagen-coated Petri dishes (2D environment), 3D collagen-PCL scaffolds (3D environment), and 3D collagen-PCL scaffolds with inhibition of integrin 72 h after treatment with HCT116-CM (scale bar = 100 µm). *p < 0.05. All values represent means ± SD, n = 5.

Heterogeneous tumor progression is caused by the “activation” response of stromal cells, including fibroblasts and endothelial cells (Figure S7), near to a growing tumor. Reciprocal reprogramming of both the tumor cells and the surrounding stromal cells can jointly drive cancer progression 42. The results demonstrated that due to their intrinsic cell plasticity, stromal cells were activated by the CM from tumor cells (Figure 3A), indicating that tumor-associated stromal cells (CAFs and TECs) were successfully generated *in vitro* from normal stromal cells (HELFs and HUVECs). Following treatment, the expression of CAF markers in fibroblasts, including α-SMA, FAP, and FSP-1, was upregulated (Figures 3B & C), and the expression 48 h after treatment was significantly different from that after 72 h (Figure 3D). Although these markers are not specifically expressed by CAFs, their high expression level can effectively help evaluate the degree of cellular activation 39. We further analyzed the activated fibroblasts by examining the expression of genes encoding tenascin C (TNC), collagen I, and TGF-β1 (Figures 3C & E), which are the protein components of the extracellular matrix and represent paracrine signals from CAFs 43, 44. The results demonstrated that the expression of CAF markers was significantly upregulated in activated fibroblasts (Figures 3C & E), thereby demonstrating that tumor CM can transform normal HELFs into CAFs *in vitro*.

We also examined activated normal HUVECs *in vitro*. The results indicated that characteristic genes (TME1, TME8, VEGF, and biglycan) were expressed in activated endothelial cells (TECs) 45, 46 (Figures 3F-I), the expression increasing with increasing duration of treatment (Figures 3F & G). Interestingly, endothelial cells underwent a significant morphological change after treatment: they displayed greater numbers of pseudopodia and an apparent tubular structure (Figure 3J). More importantly, the expression of CD144 in TECs was higher compared than in normal endothelial cells (HUVEC; Figure 3K), suggesting that activated endothelial cells may result in enhanced vascular function. This also confirms that vascular endothelial cells undergo higher levels of angiogenesis in a tumor microenvironment.

Different culture environments, e.g. 2D or 3D, can result in different degrees of cell activation. The results demonstrated that in a 3D environment, cell activation was relatively poor (Figure 3L). The expression of genes encoding adhesion-mediated proteins, including E-cadherin, N-cadherin, and integrin, was measured in cells cultured on the 3D collagen-PCL scaffold for 48 h. We observed that only integrin was significantly up-regulated (Figure 3M), indicating that the cells adhered to the 3D scaffold through integrin-mediated cell adhesion, rather than cadherin-mediated adhesion. We then evaluated how the inhibition of integrin would affect cell activation efficiency. The results indicated that the inhibition of integrin resulted in the activation efficiency of cells grown on 3D scaffolds being similar to that of cells grown in a 2D environment (Figures 3N & O). Therefore, when constructing the tumor tissues, we first activated the cells in a 2D environment and then transferred them to 3D scaffolds.

### 3D environments boost the formation of tissue network structures

**Figure 4:**
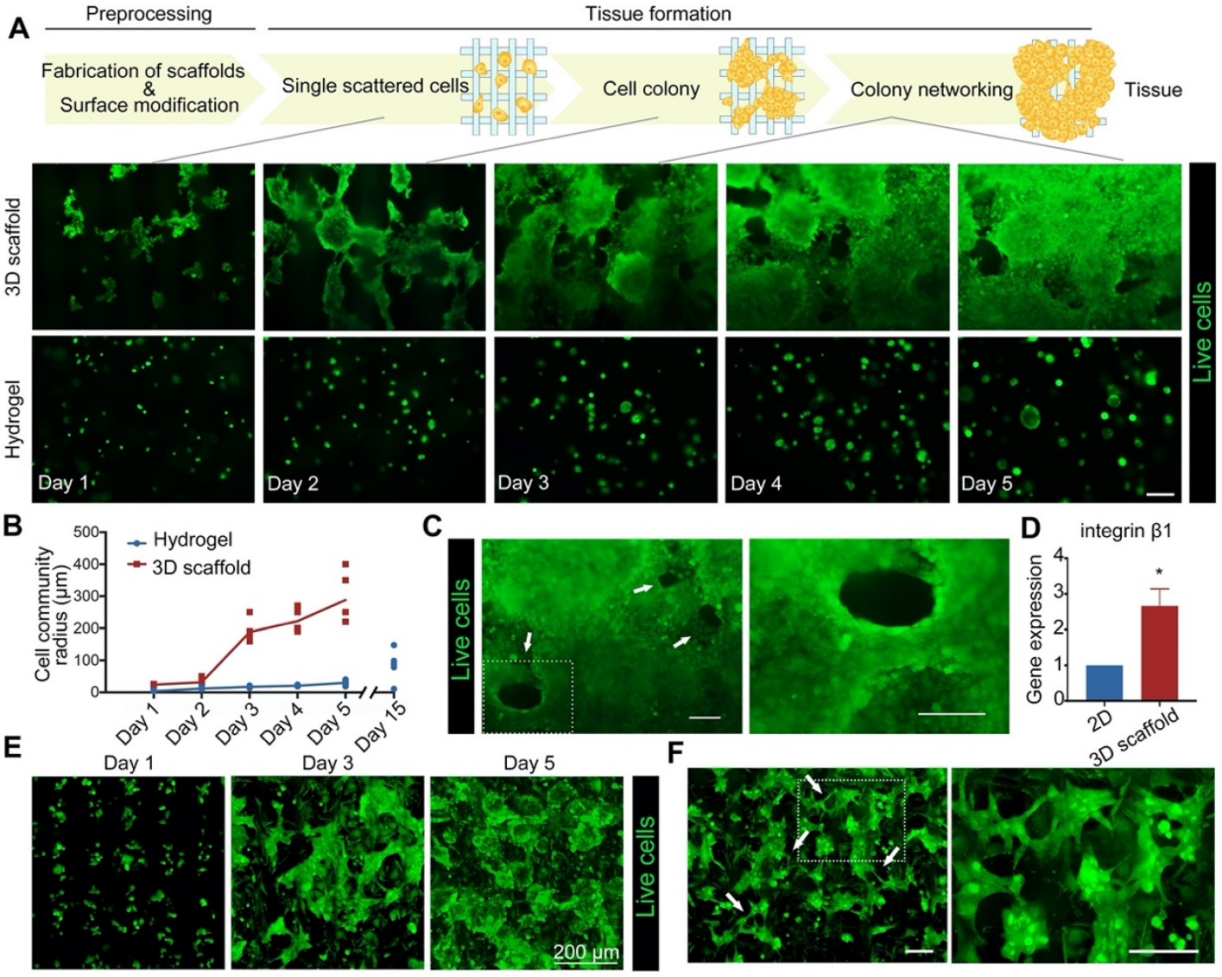
** 3D environment boosts the formation of functional networks of cells. A)** Fluorescence images of the 3D cellular networks of HCT116 cells cultured on 3D scaffolds (middle row) and hydrogel (bottom row), and a schematic illustration displaying the corresponding formation mechanism. **B)** The quantity of cells in (A) measured in the defined area, represents a change in the volume of cell aggregates during cell growth. **C)** Vascular-like cavity structure in HCT116 cells cultured on 3D scaffolds, formed at later stages of culture. **D)** Gene expression of integrin-β1 in tumor cells cultured in 2D and 3D environments. **E)** Formation of a 3D tumor tissue (containing three cell types: HCT116 cells, CAFs, and TECs). **F)** Vascular-like cavity structures in the tumor tissues, formed at the early stages of culture (green = live cells, white arrows = cavity structures, scale bar = 100 µm in (A), (C), and (F). Scale bar = 200 µm in (E)). *p < 0.05. All values represent means ± SD, n = 5.

The 3D scaffolds provide an environment suitable for cell growth. HCT116 cells cultured on 3D scaffolds formed cell communities and a spatial network (Figures 4A, 4B & S8). After one week, cells cultured on the scaffolds formed structures similar to that of a vascular cavity (Figure 4C), useful for the transport of nutrients and oxygen. However, network structures were not observed in HCT116 cells cultured in hydrogel spheroids. The network structure of tumor cells can be determined by vascular mimicry (VM), a parameter that can be used to study pre-vascularization in tumor tissues *in vitro*. The VM of tumor cells, which describes the potential of tumor cells to trans-differentiate to form a network structure on 3D scaffolds, has not been referred to in earlier models of tumors 47. The VM of tumor cells is caused by the upregulation of integrin-β1 due to matrix structure induction 47. As shown in Figure 4D, the expression level of integrin-β1 in HCT116 cells cultured on 3D scaffolds was higher than in those cultured in a 2D environment.

To investigate 3DT formation, HCT116 cells, CAFs, and TECs were co-cultured (Figure S9). It was found that the cells gradually formed multiple communities with recurrent network structures on the scaffold as the duration of culture increased. At the later stages of 3DT culture, complex network structures, the important structural basis for tumor tissues, were formed between the multicellular communities (Figure 4E). Interestingly, VM in the multicellular culture appeared earlier than that in single cellular culture of HCT116 (Figures 4F & S10), suggesting that the multicellular system may not only promote pre-vascularization, but also enhance cell communication.

### 3D tumor tissues exhibit transcriptomic characteristics of colorectal carcinoma

**Figure 5:**
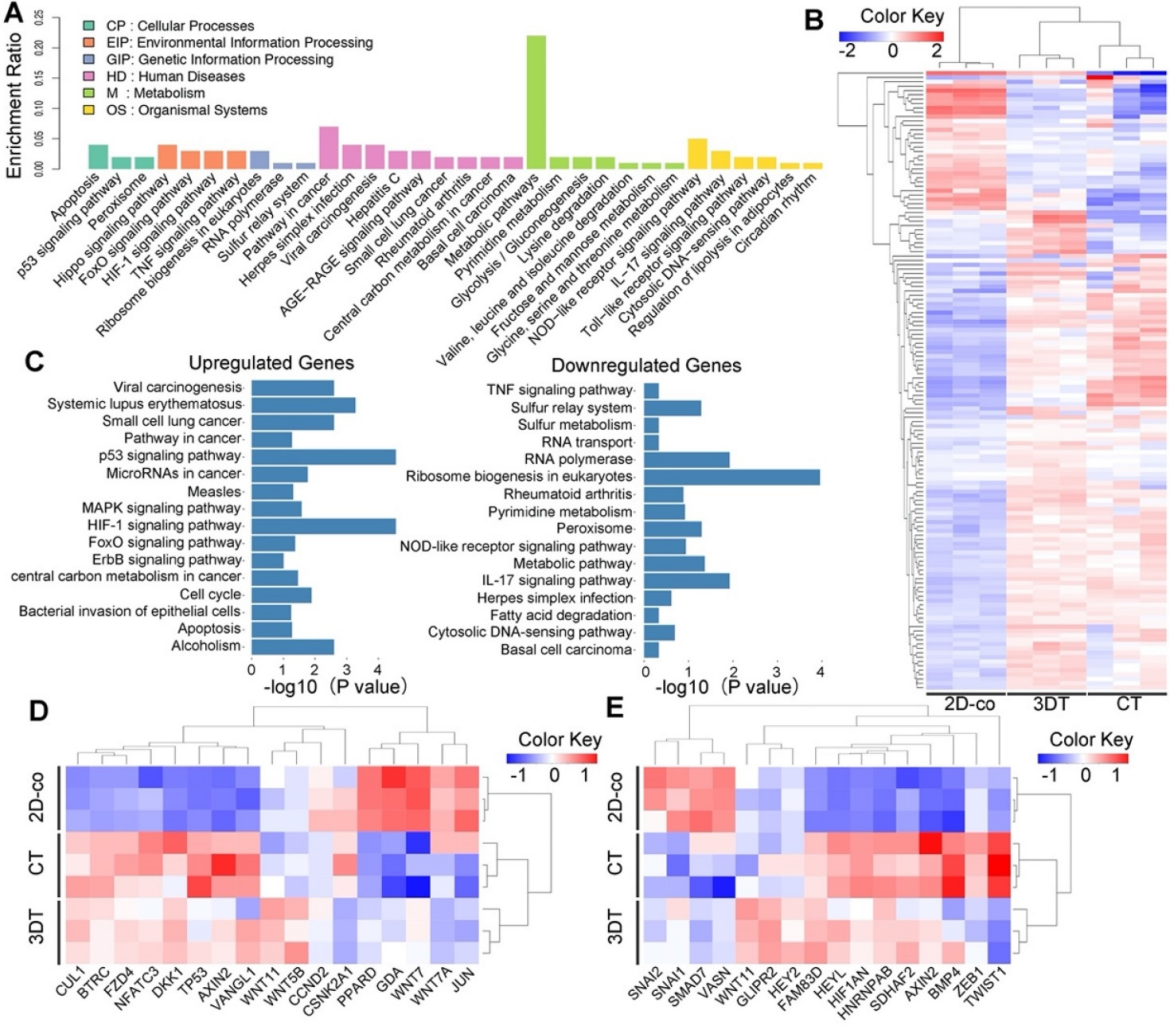
** Transcriptomic characteristics of the 3D tumor tissue. A)** Histogram displaying differential expression of genes in the KEGG pathway. The name and classification of the KEGG pathway are shown on the abscissa and enrichment ratio on the ordinate. The description of the classification is shown in the upper left corner. **B)** Clustered heatmap of 142 metabolic genes expressed in the 2D co-cultured cells (2D-co), 3D tumor tissue (3DT), and *in vivo* colorectal tumor (CT). **C)** Statistically significant upregulation and downregulation of genes in the KEGG pathway of the 3DT compared with 2D-co. **D-E)** Clustered heatmap of 17 genes in the Wnt pathway (D) and 16 genes in the EMT pathway (E) in the 2D-co, 3DT, and CT. The dendrograms shown at the top of the heatmaps (in B, D, and E) indicate similarity of gene expression patterns.

The similarity between 3DT and *in vivo* colorectal tumors were evaluated by comparison of the global transcriptomic characteristics of cells from the 3DT, 2D-co, and CT using RNA sequencing. Analysis using the Kyoto Encyclopedia of Genes and Genomes (KEGG) database indicated that the metabolic pathway had the most significant enrichment in the 3DT and 2D-co groups (Figure 5A). Unbiased hierarchical clustering was performed using a metabolic pathway gene-set consisting of 142 genes, the data of which were presented as a heatmap. The results revealed that the transcription of genes involving the metabolic pathway of the 3DT group better resembled that of the CT group, in comparison with the 2D-co group (Figure 5B). Additionally, upregulation of hypoxia-, stress- and anti-apoptosis-related signals (e.g. HIF-1, MAPK, and ErbB) and downregulation of necrosis- and inflammation-related signals (e.g. TNF and IL17) were observed (Figure 5C). These findings indicate that cells in the 3DT exhibited more physiological function than cells in the 2D-co group. Thirty-three genes involving the EMT/Wnt pathways, important in cancer research 48, 49, underwent unbiased hierarchical clustering, and presented as a heatmap. The clustering indicated that the 3DT cells displayed more similarity to *in vivo* tumor cells than those in the 2D-co group (Figures 5D & E). Thus, these results demonstrated that the functions of cells in the 3DT and *in vivo* colorectal carcinoma tissue are very similar.

### Niche cells influence the physiological state of tumor cells

**Figure 6:**
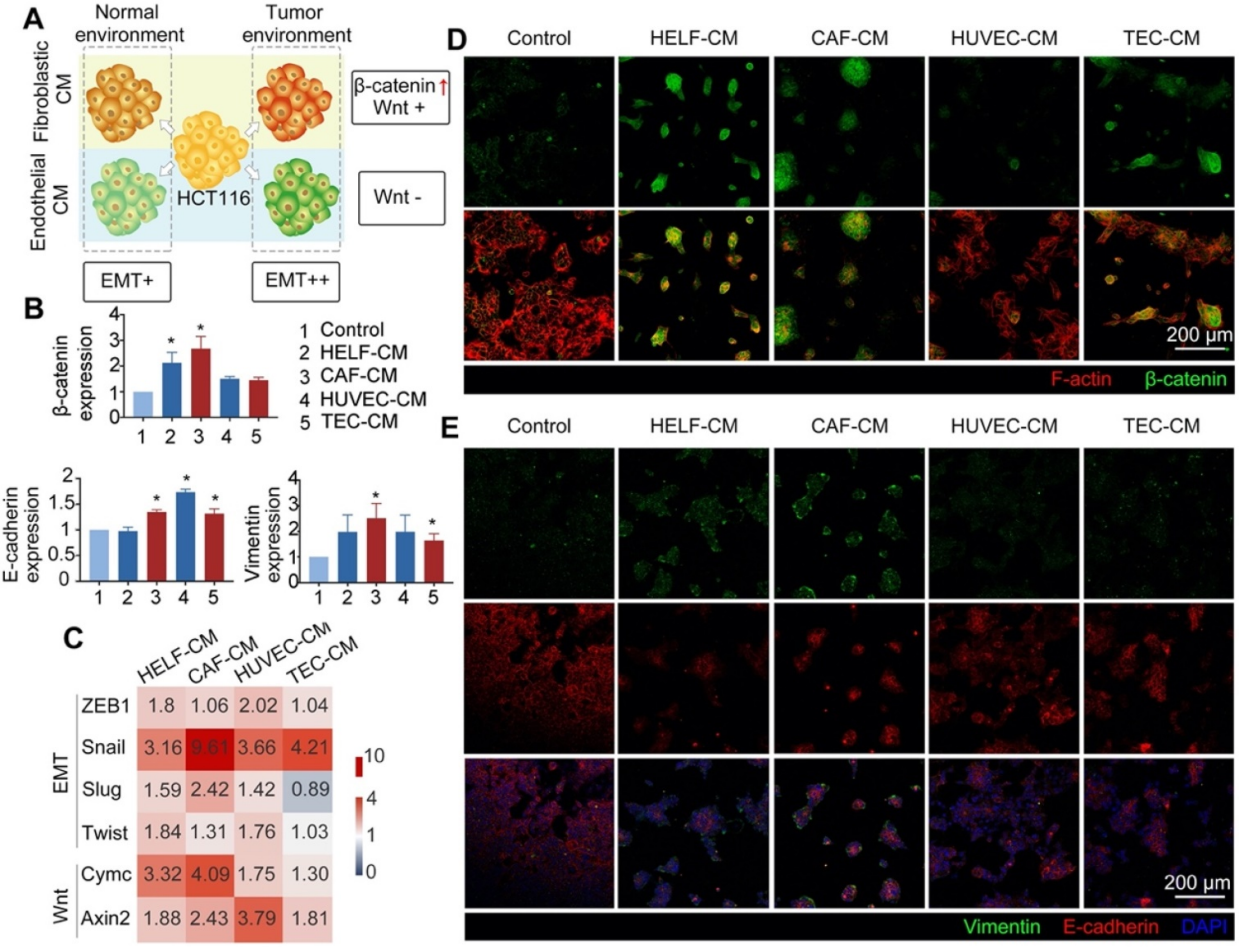
** Effect of activated stromal cells on EMT/Wnt pathways of 3D tumor tissue. A)** Schematic diagram illustrating the effect of activated stromal cells on the function of HCT116 cells. **B)** Expression of Wnt (β-catenin) and EMT (E-cadherin and Vimentin) in a variety of CM-treated cells. **C)** Heatmap indicating the fold increases relative to the control (untreated cells) of gene expression in EMT and Wnt in different CM-treated cells. **D)** Expression and location of β-catenin (green = β-catenin, red (phalloidin) = F-actin). **E)** Immunofluorescence images of EMT markers of different CM-treated tumor cells (green = vimentin; red = E-cadherin; blue (DAPI) = nuclei, scale bar = 200 µm). The control group in B, D & E was HCT116 cells. *p < 0.05. All values represent means ± SD, n = 5.

To explore the similarity between the 3DT and *in vivo* colorectal cancer tissue, and to explore whether the EMT/Wnt pathways dominated within the tumor microenvironment, CM from normal stromal cells (HELF-CM and HUVEC-CM) and activated stromal cells (CAF-CM and TEC-CM) were collected and used to treat tumor cells. The phenotypes of the tumor cells were then analyzed (Figure 6A). Analysis of EMT pathway-related gene expression indicated that the upregulation of E-cadherin was principally dependent on endothelial messaging, while the upregulation of vimentin was dependent on fibroblastic information (Figure 6B). The expression of Snail, the most affected by the matrix environment of the four classes of EMT-related transcription factor (ZEB1, Snail, Slug, or Twist), was significantly upregulated in CAF-CM and TEC-CM (Figure 6C). The situation was more complex for the Wnt pathway. Fibroblastic CM (HELF-CM and CAF-CM) and endothelial CM (HUVEC-CM and TEC-CM) displayed opposite regulatory effects. The Wnt pathway was activated in both normal and tumor-related fibroblasts in fibroblastic CM, and the expression of β-catenin was significantly up-regulated (Figure 6B). When considering C-myc and Axin2, the principal target genes of the Wnt pathway, the upregulation of C-myc was greater than that of Axin2, a Wnt suppressor gene. The upregulation of C-myc indicates that tumor cells exhibit higher proliferation. The data also indicate that the Wnt pathway and its downstream targets were activated by fibroblastic messages, and the Wnt pathway in tumor cells was fibroblast-dependent 50. However, the upregulation of Axin2 was higher than that of C-myc, while the upregulation of β-catenin was not significant. Together, these findings indicate that the Wnt pathway can be inhibited by the endothelial message, and the inhibition can be corrected by activated endothelial cells (Figure 6C).

We additionally observed Wnt activation and EMT using immunofluorescence staining. Clear cytoplasmic accumulation of β-catenin was observed in tumor cells treated with fibroblast CM (HELF-CM and CAF-CM), and the membrane localization of tumor cells treated with endothelial CM was similar to that of normal tumor cells (Figure 6D). Immunofluorescence staining for E-cadherin/vimentin demonstrated that the mesenchymal characteristics of cancer cells were significantly upregulated after treatment with CM, while a number of their epithelial characteristics were retained (Figure 6E). Interestingly, the upregulation of vimentin increased at the periphery of the tumor cell community after CAF-CM treatment, while cells in the center exhibited greater E-cadherin retention (Figure 6E). This implies that tumor-related fibroblasts have a regulatory effect on the EMT heterogeneity of tumor tissues.

### 3D tumor tissue has greater tumorigenicity and drug resistance

**Figure 7:**
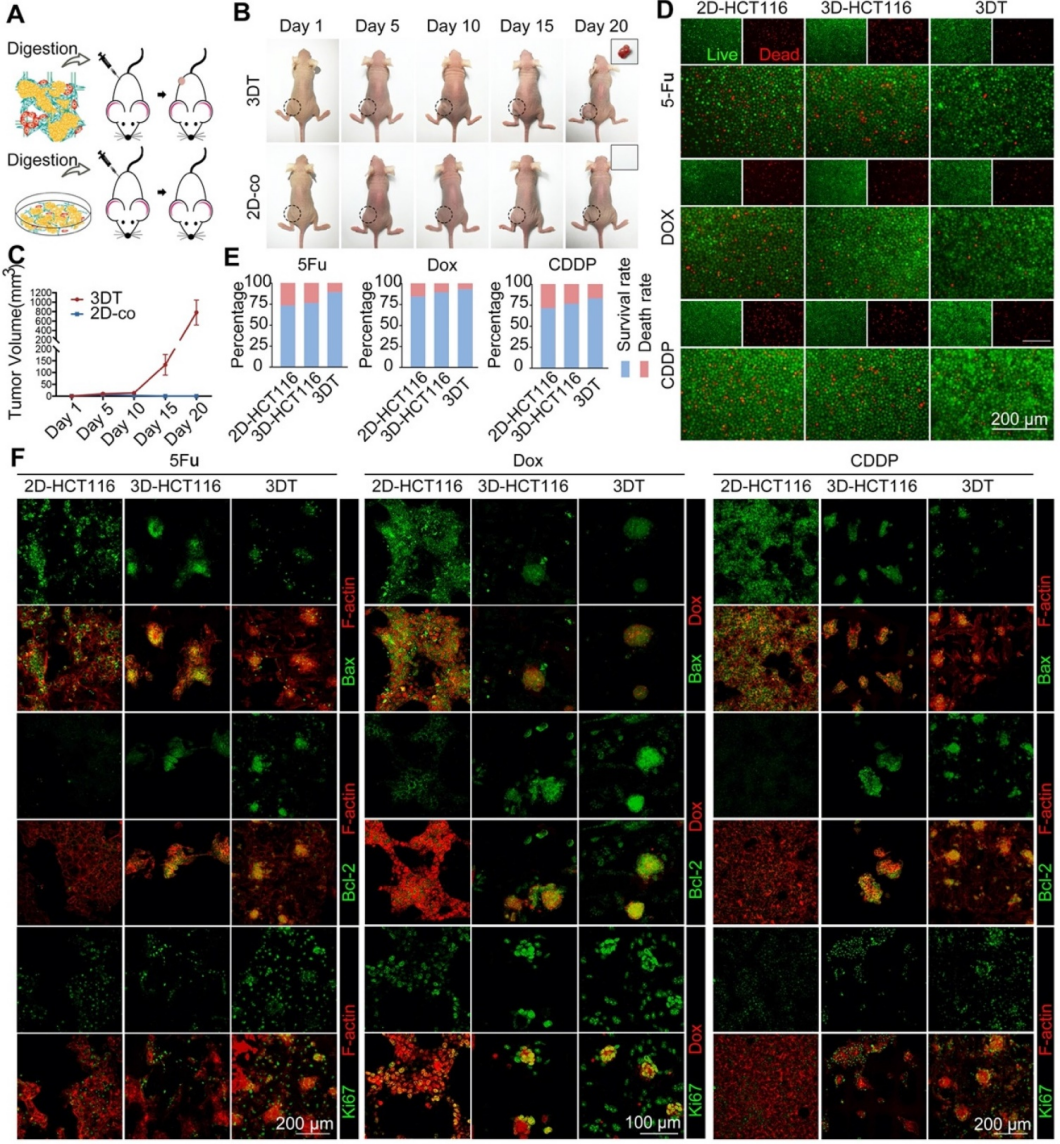
** Tumorigenicity and drug resistance of 3D tumor tissue.** A) Schematic of experimental design. B) Tumor growth images. C) Live/dead staining of tumor cells grown in different culture conditions after chemotherapy (2D-HCT116 = single 2D-cultured HCT116 cells, 3D-HCT116 = single 3D-cultured HCT116 cells, 3DT = HCT116 cells co-cultured in the 3DT group, green = live cells, red = dead cells). D) Tumor volume during growth. E) Quantification of cell growth in (C). F) Immunofluorescence staining of apoptotic marker (Bax), anti-apoptotic marker (Bcl-2), and proliferation marker (Ki67) of tumor cells grown within different culture conditions after treatment with drugs (green = Bax, Bcl-2, or Ki67, red = Dox or F-actin (phalloidin)). *p < 0.05. All values represent means ± SD, n=5.

To confirm that the 3DT displayed greater physiological function, such as tumorigenicity and drug resistance, than other *in vitro* models, we conducted *in vivo* tumorigenesis experiments and drug resistance tests. In the tumorigenesis experiment, the 3DT and 2D co-cultured cells (2D-co) were digested to ensure the numbers of seeded cells matched (Figure 7A). The results indicated that cells in the 3DT group cultured with activated stromal cells on 3D scaffolds displayed considerably greater tumorigenicity (Figures 7B & D).

The compatibility of 5-FU and platinum drugs is a gold standard for clinical colorectal cancer therapy. Dox is a commonly used chemotherapy drug that exhibits intrinsic fluorescence emission. It can be used for the convenient observation of drug transport. Therefore, 5-FU, CDDP, and DOX were chosen for the drug resistance test. According to live/dead staining (Figure 7C), the cancer cells in the 3DT group displayed a higher survival rate and lower mortality compared with single 2D- and 3D-cultured HCT116 cells. To eliminate any difference between drug penetration capability when cells were cultured in 2D and 3D environments, the viability of cells from different groups was compared. The results indicated that cells in the 3DT group displayed greater viability (Figures 7E & S11). In addition, follow-up immunofluorescence staining with antibodies directed against Bax, Bcl-2, and Ki67 additionally demonstrated that the cells in the 3DT group had greater anti-apoptosis capability (Figure 7F). No significant quantitative change was observed in stromal cells (CAFs and TECs) during drug treatment, indicating that the tumor-associated microenvironment was relatively stable (Figure S12).

## Discussion

The lack of tumor-related stromal cells in traditional cell cultures is not conducive to the study of the interactions between a tumor and its stromal environment. In this regard, patient-derived xenografts (PDXs) have proven useful models of human cancers, including colorectal cancer 51, 52, because they retain tumor histopathology (including tumor matrix components) and the overall gene expression and methylation profiles of the malignant epithelial cells of the patients. However, in addition to the long implantation cycle, which is costly and time-consuming, the success rate of PDX implantation is low 53. 3D printing offers the unique ability to create architecturally and compositionally complex biomimetic microenvironments with high reproducibility. A 3D printed tissue model recapitulates cell-matrix interactions and cell-cell communications, with vasculature and tissue heterogeneity incorporated into the model 54. Furthermore, it is expected that the model can be tailored with structures having different mechanical properties, allowing the simulation of different stages of cancer by controlling materials, and processing conditions 55. Therefore, we believe that using 3D tissue models is an alternative approach in cancer research suitable for large-scale drug tests and may represent a promising paradigm for establishing personalized medicine.

TME is important for disease progression and drug response, and recent advances in 3D culture techniques coupled with the ability to independently manipulate various microenvironmental factors have enabled the real-time study of tumor tissues. Herein, we fabricated an *in vitro* 3D colorectal cancer tissue model using 3D-printed scaffolds and activated stromal cells to provide a microenvironment that simulated the metabolism and physiological characteristics of *in vivo* tumor tissues. The results demonstrate that the 3D-printed collagen-PCL scaffolds provided multi-functional support for cells while promoting their physiological function, such as growth, adhesion, stemness, pre-vascularization, and matrix remodeling. Through the use of activated stromal cells, it was possible to generate a TME that mimicked an *in vivo* TME while properly restoring physiological tumor signaling, such as EMT/Wnt pathways. The system established in this way displayed great flexibility as the 3D structures, in addition to the extracellular microenvironment, could be custom-tailored, thus, rendering it suitable as a platform for the continuous monitoring of cancer growth or drug screening.

PCL has been used as a carrier in tissue engineering because of its biocompatibility and slow degradation 56, 57. The good solubility of PCL enables high processability and flexibility to control the fiber diameter and pore-size of scaffolds produced by the E-jet 3D printing system. Collagen is an important component of the tumor matrix, which is closely linked to tumor growth and invasion 58, and playing a crucial role in conveying regulatory signals from the extracellular space to the cells 59. After coating with collagen, the scaffolds were capable of recruiting cells. The number of attached cells on the collagen-coated scaffolds was much greater than that observed on uncoated scaffolds. A scaffold that has a micropore diameter of more than 100 μm facilitates greater cell penetration 60. The 3D-printed scaffolds developed in the present study met this requirement and thus better promoted the adhesion and proliferation of cancer cells *in vitro*. Cells on the scaffolds were able to spread in three-dimensions, thus the exponential growth phase was achieved rapidly. In addition, the orderly fibers of the scaffold could be severed to produce an aligned ECM structure, which promoted cell migration 61. More importantly, the results demonstrated that 3D scaffolds promoted the pre-vascularization and proliferation of stem cell tissues.

In previous studies, it has been shown that a co-culture of tumor cells with stromal cells (such as endothelial cells and fibroblasts) can promote tumor angiogenesis 62, 63. Here, we demonstrated that 3D scaffolds promote the vascular mimicry of stromal cells, possibly induced by the morphology of the scaffolds, representing great significance in establishing a 3DT. In addition, stem cell behavior and cell differentiation are heavily influenced by local *in vivo* biomechanical forces, such as those resulting from interactions with the extracellular matrix 64. Tumor cells grown on the 3D scaffolds had a stem cell-like phenotype as a result of the 3DT construction. A significant feature of the 3DT as a tumor model is the use of activated cells because the introduction of a physiologically-related ECM remains fundamentally challenging for *in vitro* tumor models 65. The conventional use of Matrigel has many limitations, such as being unable to simulate *in vivo* growth factors and signal transduction 18. However, the co-culture of stromal cells is a possible solution to this limitation. The expression of tumor-related genes in tumorous stromal cells and normal stromal cells is substantially different, and as a result, the progression and prognosis of tumors can vary significantly 66-68. We demonstrated that the activation of stromal cells using tumor CM caused them to express genes that were markers of tumor-associated stromal cells. TGF-β is the most important factor that induces EMT, a process that promotes tumor metastasis 69. The tumor-related ECM components TNC and biglycan can regulate tumor angiogenesis 43, 46. We measured the expression of these genes. However, to gain insight into the oncological process, real-time imaging combined with single cell profiling should be conducted in future.

The traditional generation of tumor tissues using hydrogels allows tumor cells to form spheroids, but not a network structure. The network structure between cells is important because it is closely related to VM, the trans-differentiation potential of tumor cells to form network structures in ECM 47. The tumor cells lining these networks do not express endothelial surface markers, such as CD31 70, 71. Our results confirmed that tumor cells grown on the 3D scaffolds formed a vascular-like ring structure with a distinct lumen, especially in the later stages. VM was observed at the early stage of 3D tumor tissue formation, indicating that the 3D scaffolds are suitable for the generation of tumor tissues.

The activated niche in the 3DT plays a key role in the recovery of signal transduction *in vivo* 72. Gene expression clusters were plotted following transcriptome sequencing analysis. The results indicated that the gene expression patterns of the 3DT resembled that of *in vivo* colorectal tumors, especially for genes involving the signaling of metabolic pathways, in addition to other tumor-related signaling pathways, such as the EMT and Wnt pathways, which dynamically regulate tumor progression, and which are regulated by the tumor microenvironment 73, 74. Treatment with CM revealed that activated cells had a significant effect on the physiological activities of tumor cells. Our results demonstrated that the activation of Wnt in tumor cells is fibroblast-dependent, consistent with previous reports 74. Endothelial cells exhibited clear downstream inhibition. However, activated endothelial cells were able to address this issue, and canonical the Wnt/β-catenin pathways appear to be the mechanism involving TEC-derived proangiogenic action 75.

To assess the physiological activity of the 3DT and its efficacy as a drug screening platform, tumorigenic experiments and drug resistance tests were conducted. The results indicated that the 3DT displayed greater tumorigenic capability and drug resistance compared with 2D/3D co-cultured normal stromal and tumor cells. The expression of apoptosis and proliferation proteins and the survival rate of cells in 3DTs were significantly different to those in the 2D-co group. The 3DT has many advantages over other systems in which other cell types are grown within the same dimensional settings, although the 3D scaffolds have been shown to influence drug delivery 76, 77. These findings demonstrate that the developed 3DT could replicate the *in vivo* physiological and pathological conditions of cancer tissues*,* and therefore able to be used as a model for clinical therapies.

## Conclusions

In summary, we developed a laboratory controllable, *in vitro* 3D tumor tissue model with a physiological environment using 3D printing technology and cell remodeling. Colorectal cancer cells, cancer-associated fibroblasts, and tumor-associated endothelial cells were co-cultured on the scaffolds. The 3D-printed collagen-PCL scaffolds provided multi-functional support for cells while promoting their physiological function. The use of activated stromal cells was found to result in the generation of a TME that mimics an *in vivo* TME, properly restoring physiological tumor signaling, such as the EMT/Wnt pathways. The developed 3D tumor model helped restore the physiological metabolism of the tumors and other functions, including tumor malignant transformation. It also had flexible physiological function, making it applicable to other pathologies, and able to be used in many other future cancer studies. Thus, the present model of a tumor may be combined with single-cell imaging, sequencing technology, or gene-editing tools to develop other translatable tissue models to provide a deeper understanding of cancer matrix interactions and their impact on tumor cell response to therapies.

## Supplementary Material

Supplementary figures and tables.
