# Supplementary material for: 3D printed in vitro tumor tissue model of colorectal cancer
Source: Theranostics. 2020 Oct 26;10(26):12127–43. doi: 10.7150/thno.52450 (PMC7667682; doi:10.7150/thno.52450)
Supplement: Supplementary file 1 — Supplementary figures and tables. [file thnov10p12127s1.pdf]

Supporting Information

3D printed *in vitro* tumor tissue model of colorectal cancer

Haoxiang Chen[1,2*], Yanxiang Cheng[3*], Xiaocheng Wang[1], Jian Wang[1,2], Xuelei Shi[1,2], Xinghuan Li[1], Weihong Tan[1], Zhikai Tan[1,2]

* The authors contributed equally to the work.

Corresponding author: Dr. Zhikai Tan,

Email: tanzk@hnu.edu.cn;

1. College of Biology, Hunan University,

Changsha, Hunan, 410082, China.

2. Shenzhen Institute, Hunan University,

Shenzhen, Guangdong, 518000, China.

3. Department of Obstetrics and Gynecology,

Renmin Hospital, Wuhan University,

Wuhan, Hubei, 430060,China

Table S1. Sequences of primers used for qRT-PCR

Gene	Forward primer	Reverse primer
GAPDH	GTCAAGGCTGAGAACGGGAA	AAATGAGCCCCAGCCTTCTC
β-actin	TGGCACCCAGCACAATGAA	CTAAGTCATAGTCCGCCTAGAAGCA
α-SMA	CAGGGCTGTTTTCCCATCCAT	ACGTAGCTGTCTTTTTGTCCC
FAP	ACGGCTTATCACCTGATCGG	AATTGGACGAGGAAGCTCATTT
FSP1	GATGAGCAACTTGGACAGCAA	CTGGGCTGCTTATCTGGGAAG
Collagen I	ATCAAGGTCTACTGCAACAT	CAGGATCGGAACCTTCGCT
TGF-β1	ACTGCAAGTGGACATCAACG	TGCGGAAGTCAATGTACAGC
Tenascin-C	CCTTGCTGTAGAGGTCGTCA	CCAACCTCAGACACGGCTA
TEM 1	TCGAGTGTTATTGTAGCGAGGGACATG	AGGTGGGCTCCGGGTAGGGTAT
TEM 8	CGGATTGCGGACAGTAAGG	GCCAGAACCACCAGAGGAG
VEGF	TTGCCTTGCTGCTCTACCTCCA	GATGGCAGTAGCTGCGCTGATA
Biglycan	AGGAGGCGGTCCATAAGAAT	AGGGTTGAAAGGCTGGAAAT
E-cadherin	CTGAGAACGAGGCTAACG	TTCACATCCAGCACATCC
Vimentin	GGTGGACCAGCTAACCAACGA	TCAAGGTCAAGACGTGCCAGA
ZEB-1	TTCAAACCCATAGTGGTTGCT	TGGGAGATACCAAACCAACTG
Snail	CCTCCCTGTCAGATGAGGAC	CCAGGCTGAGGTATTCCTTG
Slug	GGGGAGAAGCCTTTTTCTTG	TCCTCATGTTTGTGCAGGAG
Twist	GGAGTCCGCAGTCTTACGAG	TCTGGAGGACCTGGTAGAGG
C-myc	CCTCCACTCGGAAGGACTATC	TTGTGTGTTCGCCTCTTGAC
Axin2	CCTGCCACCAAGACCTACAT	TTTCCTCCATCACCGACTG
CD133	TTACGGCACTCTTCACCT	TATTCCACAAGCAGCAAA
Ki67	CTTTGGGTGCGACTTGACG	GTCGACCCCGCTCCTTTT
Integrin-β1	TGATTGGCTGGAGGAATGTTA	GTTTCTGGACAAGGTGAGCAA

Table S2. Abbreviations used in the text

Abbreviation	Full name
TEM	Tumor microenvironment
CAF	Cancer-associated fibroblasts
TEC	Tumor-associated endothelial cells
ECM	Extracellular matrix
3D	Three-dimensional
2D	Two-dimensional
PCL	Polycaprolactone
CM	Conditional medium
3DT	3D tumor tissue
2D-co	Two-dimensional co-culture
CT	Cancer tissue (control group in Fig.5)
HCT-CM	Conditional medium from HCT116
HELF-CM	Conditional medium from HELF
HUVEC-CM	Conditional medium from HUVEC
CAF-CM	Conditional medium from CAF
TEC-CM	Conditional medium from TEC
2D-HCT116	Two-dimensional cultured HCT116
3D-HCT116	Three-dimensional cultured HCT116
5Fu	5-Fluorouracil
CDDP	Cisplatin
Dox	Adriamycin

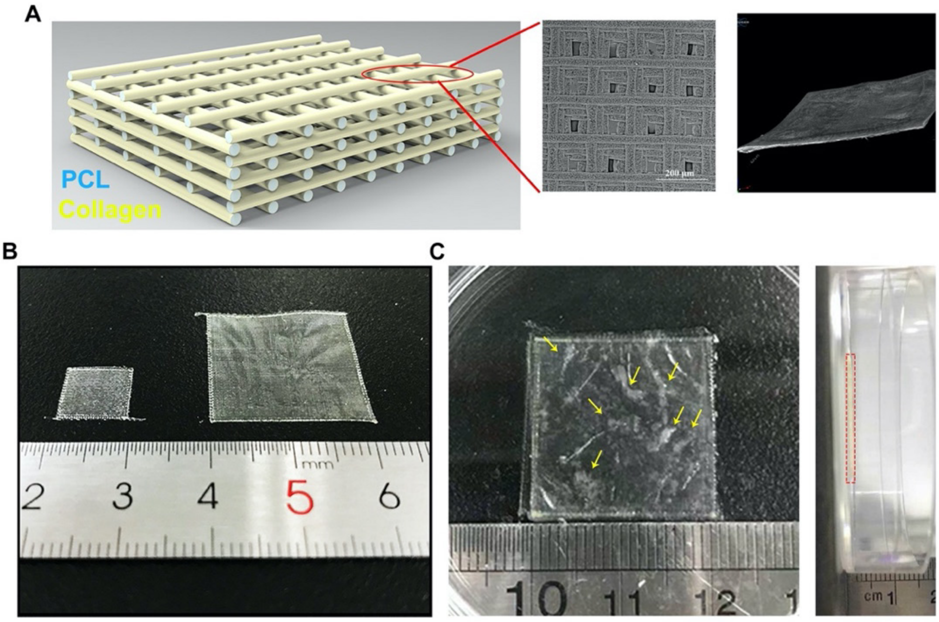

Figure S1. 3D-printed scaffolds. A) Schematic illustration and the corresponding SEM and micro-CT images of the scaffolds (scale bar = 200 μm). B) Photograph of uninoculated scaffolds with different sizes. C) Photographs of the scaffold inoculated with cells (left: top-view, right: side-view), the yellow arrow indicates the aggregation of cells in the scaffold.

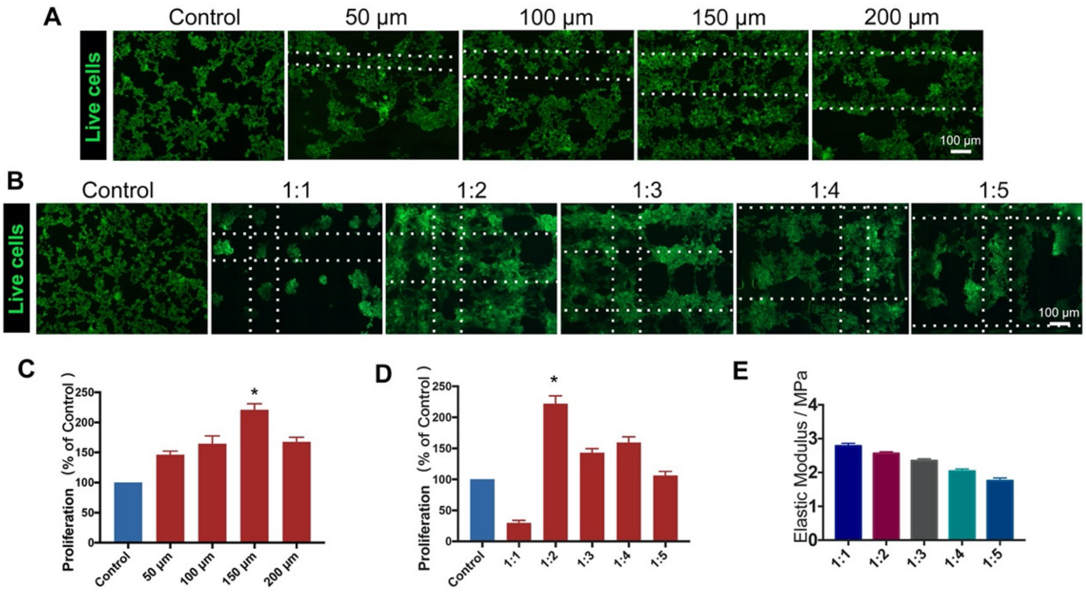

Figure S2. Cell growth on varied 3D scaffolds. A) Effect of different fiber spacings of scaffolds on growth of HCT116 cells. B) Effect of different pore sizes of scaffolds on growth of HCT116 cells. C) Quantification of cell proliferation from (A). D) Quantification of cell proliferation from (B). (green = live cells, white dotted lines indicate the center of the scaffold fibers, scale bar = 100 μm). E) Elastic Modulus of

scaffolds with different pore sizes. *p < 0.05. All values represent means ± SD, n = 5.

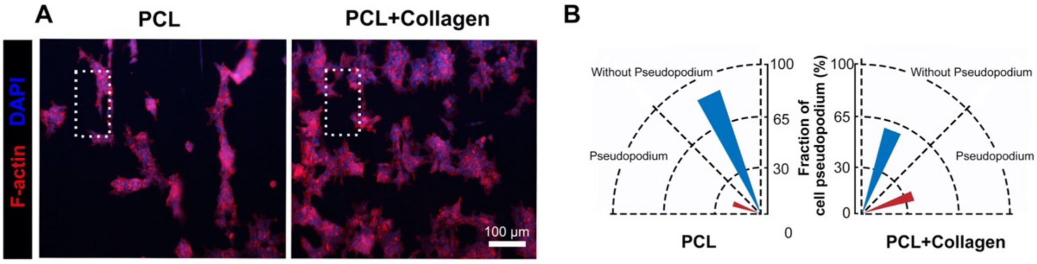

Figure S3. Adhesion of HCT116 cells to scaffolds with or without collagen coating. A) Effect of collagen on the formation of cell pseudopodia. Red = F-actin, blue (DAPI) = nuclei, white dotted rectangles represent the rectangular holes of scaffolds, scale bar = 100 μm. B) Quantification of cell pseudopodium from A.

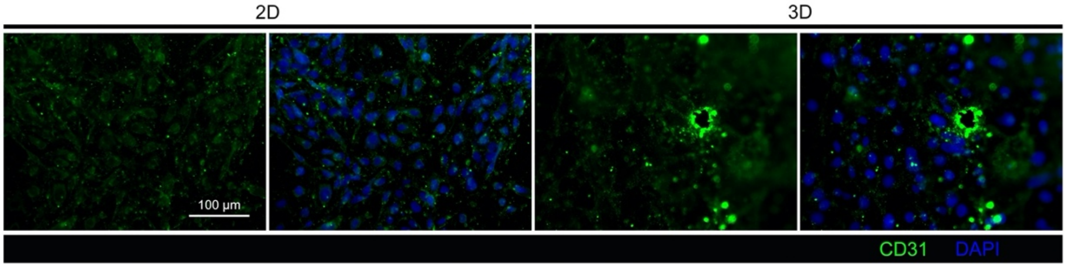

Figure S4. Immunofluorescence staining of CD31 (green) in HUVEC grown in 2D and 3D environments. Scale bar = 100 μm.

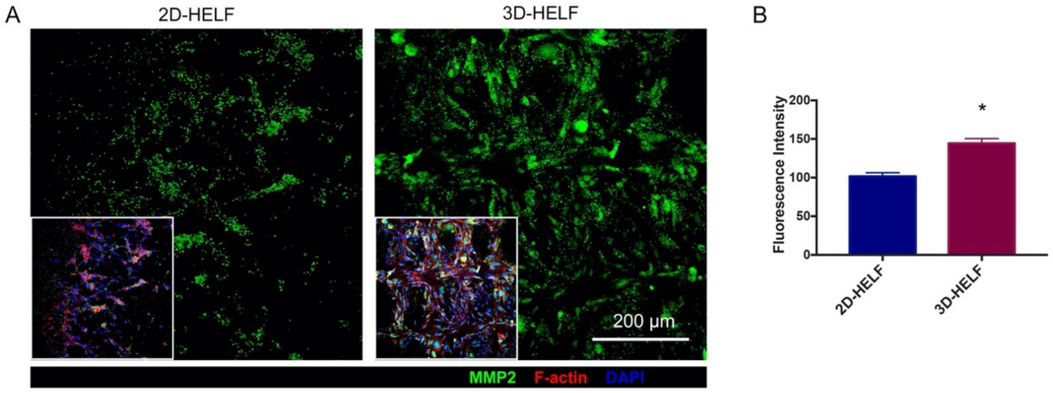

Figure S5. 3D scaffolds promote MMP2 expression of HELF. A) Immunofluorescence staining of MMP2 (green) in HELF grown in 2D and 3D environments (scale bar = 200 μm). B) Quantification of fluorescence intensity from A. *p < 0.05. All values represent means ± SD, n = 5.

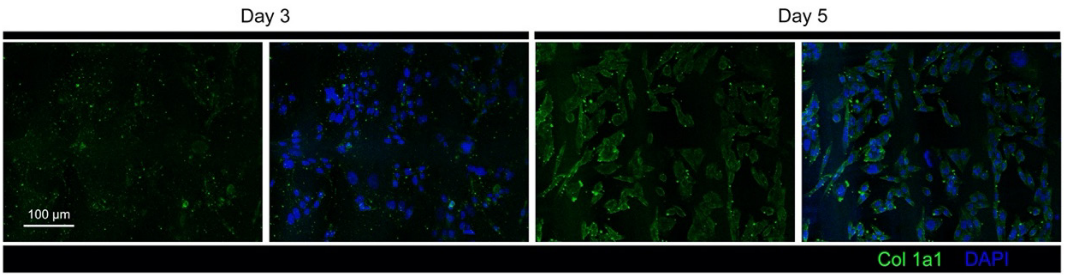

Figure S6. Collagen deposition on 3D scaffolds. The immunostaining of 3D cultured HELF for 3 and 5 days (collagen (Col 1a1), green; nucleus (DAPI), blue). Scale bar = 100 μm.

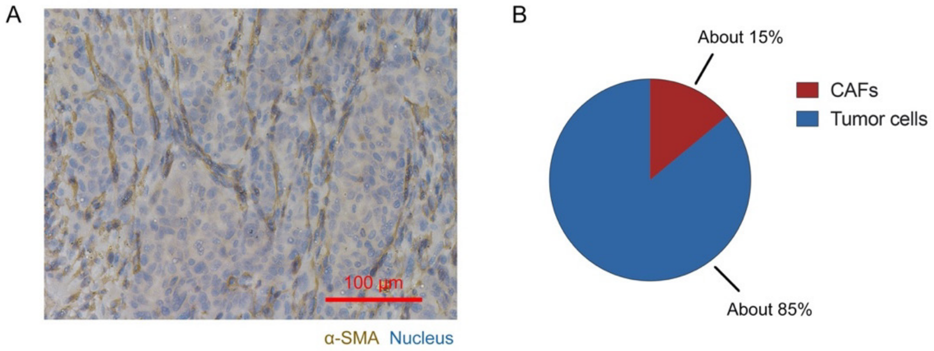

Figure S7. Characterization and quantification of CAFs in tumor tissues. A) immunohistochemical images of tumor slices. The brown color represents positive staining of α-SMA. B) Quantification of cells from A.

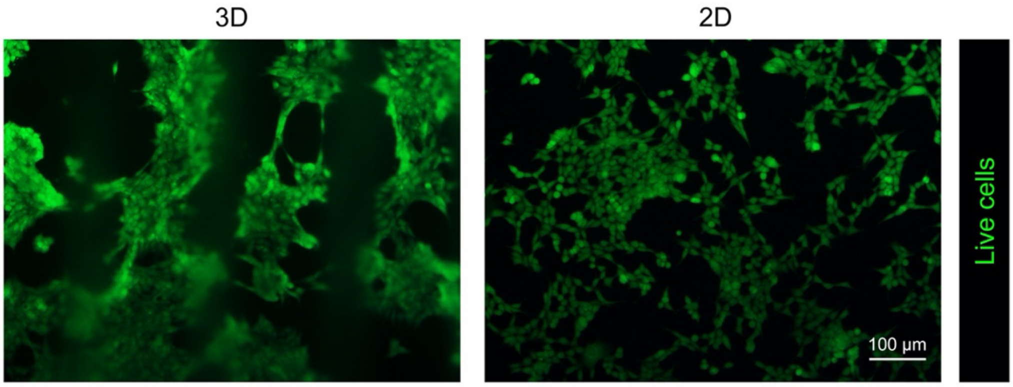

Figure S8. Morphological comparison between 3D scaffold cultured and 2D cultured tumor cells after 2 days. Green = live cells.

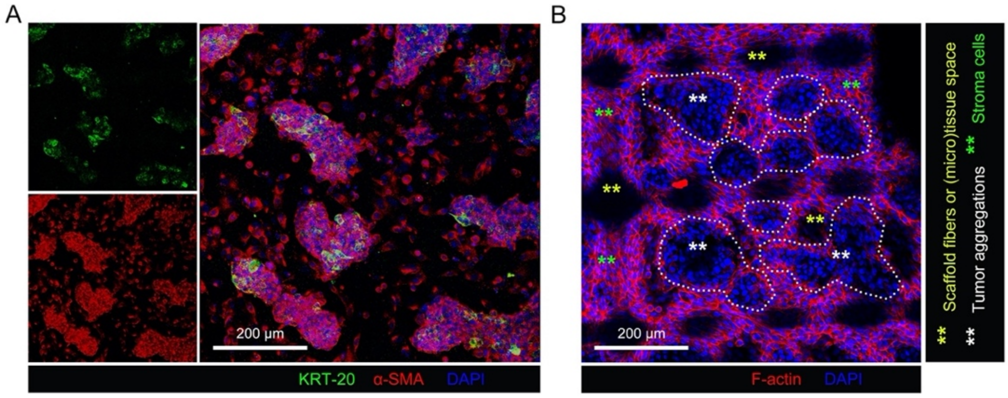

Figure S9. Immunostaining images of the co-culture system. A) The immunostaining of tumor cells (KRT-20, green) and stroma cells (α-SMA, red). B) Morphological features of cell aggregates. The white dotted line indicates the boundary of the tumor cluster. Scale bar = 200 μm.

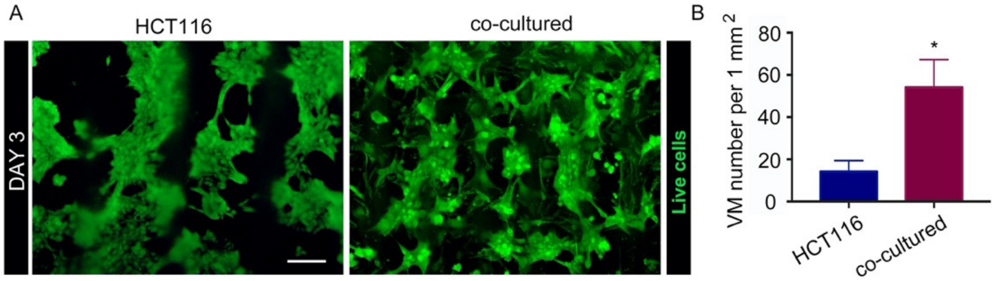

Figure S10. Comparison of vascular mimicry of single cultured and co-cultured cells. A) Morphological characteristics of HCT116 cells on the third day. Scale bar = 100 μm. B) Quantification of vascular mimicry from A. *$p < 0.05$. All values represent means ± SD, n = 5.

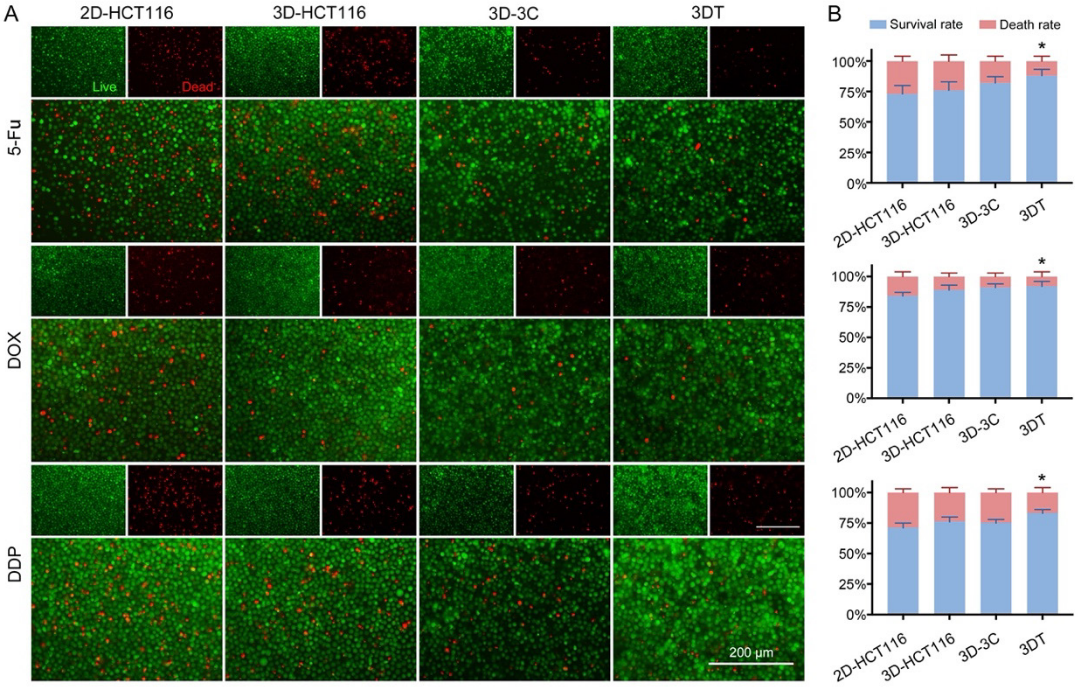

Figure S11. Drug resistance of bionic tumor tissues. A) Live/Dead staining of tumor cells grown under different culture conditions after chemotherapy (2D-HCT116 = single 2D-cultured HCT116 cells, 3D-HCT116 = single 3D-cultured HCT116 cells, 3D-3C = HCT116 cells 3D co-cultured with HELF/HUVEC, 3DT = HCT116 cells 3D co-cultured with CAF/TEC, green = live cells, red = dead cells). B) Quantification of cell growth from A. *$p < 0.05$.

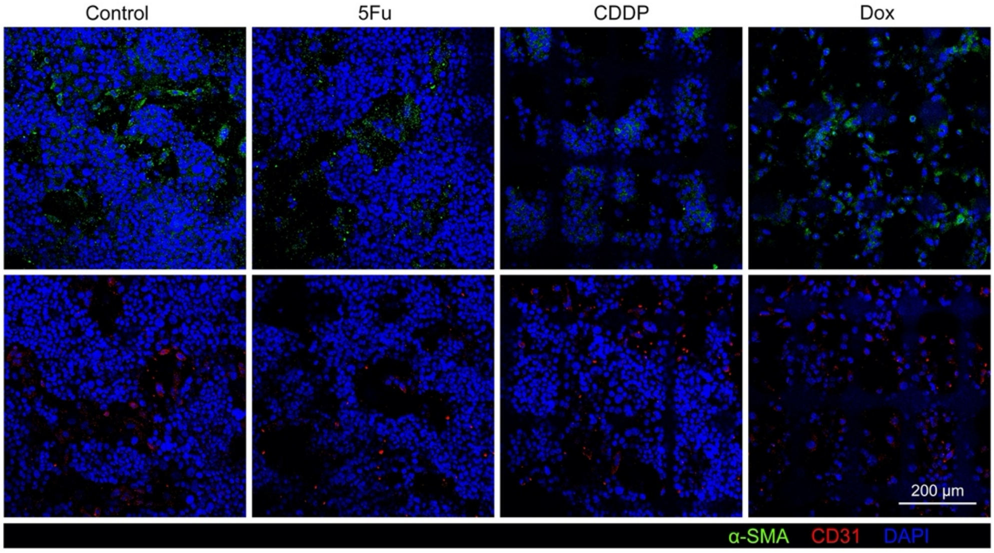

Figure S12. Immunofluorescence images of stromal cells after 48 hours of drug treatment. The treatment was performed on the tumor tissue model. The drugs used were 5FU, CDDP, and DOX; untreated group was used as the control. α-SMA represents CAFs (green) and CD31 represents TECs (red). Scale bar = 200 μm.
